# Commentary: Age Differentiation within Gray Matter, White Matter, and between Memory and White Matter in an Adult Life Span Cohort

**DOI:** 10.3389/fnagi.2019.00093

**Published:** 2019-04-18

**Authors:** Marit F. L. Ruitenberg, Kaitlin E. Cassady

**Affiliations:** ^1^Department of Experimental Psychology, Ghent University, Ghent, Belgium; ^2^Department of Psychology, University of Michigan, Ann Arbor, MI, United States

**Keywords:** aging, dedifferentiation, brain structure, cognition, compensation strategies

Healthy aging is associated with changes in cognitive, perceptual, and motor abilities, as well as changes in brain properties. Studies have shown that individual differences within such abilities and brain properties become increasingly correlated in older age: this is known as the de-differentiation hypothesis of aging. Using measures of brain *function*, prior studies have shown de-differentiation in neural representations of cognitive (Carp et al., 2010; Park et al., 2010), perceptual (Park et al., 2004; Carp et al., 2011b), and motor functions (Carp et al., 2011a; Bernard and Seidler, 2012). Older adults also show lower within-network and higher between-network functional connectivity compared to younger adults, indicative of de-differentiation of functional networks (Damoiseaux, 2017; Cassady et al., 2019). Less is known about whether the covariance within *structural* brain properties changes with older age. Furthermore, it is unclear whether aging affects the strength of brain-behavior associations. Recently, de Mooij et al. (2018) addressed these open questions and assessed structural (gray/white matter) brain properties and cognitive abilities in subjects aged 18–88 years. Results of their cross-sectional study showed that older age was associated with lower covariance *within* both GM and WM, suggesting that these structural brain properties become more specific (i.e., differentiated) with age. For cognitive abilities, covariance was independent of age, suggesting that individual differences within this domain are stable across the lifespan. Moreover, results revealed an age-related change in brain-behavior associations, in that older age was associated with decreased covariance *between* hippocampal WM connectivity and memory performance.

These findings have implications for our understanding of de-differentiation and theories on the neurocognitive effects of healthy aging. The observation that structural properties of individual brain regions become more distinct with older age suggests that aging is associated with differentiation of brain structure. An explanation may be that some brain regions are more sensitive to effects of aging than others. Alternatively, (learning) experiences in life may protect from changes in some brain areas and/or accelerate changes in others. A challenge in interpreting the results in light of the de-differentiation hypothesis is the discrepancy between these structural findings and prior functional findings. The observation that brain structural covariance decreases with age contrasts results from functional studies, which as aforementioned showed that aging is associated with increasing covariance (i.e., de-differentiation) in brain activation patterns and resting-state networks. Additionally, functional de-differentiation typically refers to age-related increases in the covariance of activation patterns *within* an individual (e.g., older adults show increasingly similar patterns for left and right hand movements), whereas de Mooij et al. (2018) assessed structural and cognitive differentiation *between* people (e.g., older adults doing poorly on memory tests also do poorly on language tasks). Bridging these two fields, we suggest that localized structural changes may actually drive the recruitment of other (more intact) areas with older age to compensate for anatomical deterioration. This would result in increasingly overlapping recruitment patterns for different behaviors with age and could explain why functional patterns across different tasks become more similar. Such additional recruitment of brain areas as a compensatory mechanism for structural changes fits theories on age-related neurocognitive changes that propose adaptive strategies to maintain relatively stable performance with age (Cabeza et al., 2018). Compensation could involve functional plasticity, where cognitive performance is accompanied by functional alterations in the brain (Greenwood, 2007), or cognitive reserve, where additional recruitment reflects cognitive processes or strategies that are engaged to cope with structural declines (Whalley et al., 2004). However, a caveat in establishing the here proposed relationship between functional and structural results is that as aforementioned these measures are determined differently (within vs. between individuals, respectively), which poses a challenge for integrating these two lines of research.

While de Mooij et al.'s (2018) results provide great insight into structural (re)organization during the lifespan and its relationship with cognitive function, we see some limitations that require cautious interpretation of the findings. First, the study involves a limited set of cognitive and neural variables, leaving open the question of whether these findings can be extended to other cognitive functions and other indices of brain structure. In a previous study, Cox et al. (2016) used a cross-sectional approach to examine various WM measures and observed increased covariance of WM microstructure across brain tracts with older age in 4 out of their 5 measures. These measures included fractional anisotropy as used by de Mooij et al., but also others such as mean diffusivity which proved to be most sensitive to age-related changes. Cox et al. (2016) thus provide strong support for robust de-differentiation of structural WM networks with aging. While de Mooij et al.'s (2018) findings contrast this notion, a notable strength is that they used multiple strategies to analyze age-related covariance changes. The observation that the results converged to the same conclusion suggests that these findings may be quite robust, too. The generalizability of the present findings should be further investigated in future studies.

A second limitation is that the segmentation approach may not be sensitive enough to detect subtler changes that occur with aging. For example, in the GM analysis the frontal lobe is considered as a single region, although prior studies have shown that different sub-regions of the frontal lobe that underlie distinct functions are differently affected by aging (e.g., Tisserand et al., 2002; Resnick et al., 2003). Moreover, the fact that the frontal lobe loaded onto each of the three modeled GM factors (de Mooij et al., 2018, Figure 2) suggests that indeed the brain may not be segmented very precisely, which could artificially inflate the chances of finding significant correlations. Evaluating sub-regions could be especially important when investigating changes in covariance related to age-related neurodegenerative disorders that affect specific regions/networks in the brain.

Overall, de Mooij et al. (2018) demonstrate that aging is not systematically associated with either increases or reductions of differentiation, but that the effects of aging are more complex and may differ for cognitive abilities and brain structural properties. Moreover, their study suggests that the strength of brain-behavior associations may change with age. While these findings thus elucidate the (absence of) differentiation in cognitive abilities and structural brain properties in healthy aging, future studies should also evaluate changes related to pathological aging. Furthermore, future work could consider individual differences in dopaminergic gene profiles predictive of cognitive and motor function (Hupfeld et al., 2018) and investigate how these interact with aging. Longitudinal designs may help to investigate these issues and adjudicate whether the patterns of age-related changes observed by de Mooij et al. (2018) are related to adaptive reorganization or cognitive reserve. In addition, such designs would allow determining the relative contribution of the degree of covariance vs. the amount of change in structural brain properties to changes in cognitive abilities with older age.

## Author Contributions

MR and KC equally contributed to the conception of the work. MR drafted the first version of the manuscript. KC critically edited the manuscript. Both authors read and approved the final version of the manuscript.

### Conflict of Interest Statement

The authors declare that the research was conducted in the absence of any commercial or financial relationships that could be construed as a potential conflict of interest.
